# The Epidemiology of Human Papillomavirus (HPV) Infections in Poland in the Light of the Nationwide HPV Vaccination Program for Children Aged 12–13 and Updated HPV DNA Detection Guidelines

**DOI:** 10.3390/ijms27031434

**Published:** 2026-01-31

**Authors:** Mateusz Sztuka, Agnieszka Jeleń, Adrian Krygier, Dagmara Szmajda-Krygier, Ewa Balcerczak

**Affiliations:** 1 Department of Pharmaceutical Biochemistry and Molecular Diagnostics, Medical University of Lodz, Muszynskiego 1, 90-151 Lodz, Poland; matisztu@gmail.com (M.S.); adrian.krygier@umed.lodz.pl (A.K.); dagmara.szmajda@umed.lodz.pl (D.S.-K.); ewa.balcerczak@umed.lodz.pl (E.B.); 2 Laboratory of Molecular Biology, Medical Laboratory Synevo Lodz Sp. z o.o., Krakusa 28, 93-515 Lodz, Poland

**Keywords:** high-grade squamous intraepithelial neoplasia, high-risk HPV, HPV genotyping, human papillomavirus infection, low-grade squamous intraepithelial neoplasia, low-risk HPV, retrospective analysis

## Abstract

Many countries have introduced HPV screening and vaccination programs to reduce the burden of cervical cancer. In Poland, before 2023, HPV vaccination was available only on an individual, non-universal basis, using all types of vaccines, while in 2023, a nationwide vaccination program for boys and girls aged 12–13 years was introduced alongside updated screening guidelines. This retrospective study analyzed 2296 HPV-positive test results obtained from adult patients in Poland, including demographic data, HPV genotypes distribution, infection intensity, and cytological findings. HPV genotyping was performed using the Anyplex™ II HPV28 assay. HR-HPV genotypes accounted for 64.53% of all detected infections, with the highest prevalence observed in individuals aged 26–35 years of both sexes. HPV-18 was significantly more frequently in women (*p* = 0.0430), whereas HPV-53 predominated in men (*p* = 0.0030). Men more often presented low-intensity infections, while women showed higher viral load. Multigenotypic infections occurred in 46.5% of cases, particularly among younger patients (*p* < 0.001), and were significantly associated with LSIL changes in cytology. The HSIL type correlated most strongly with HPV-16 (*p* < 0.001). These findings confirm the high burden of HR-HPV infections in the Polish adult population and provide an essential epidemiological baseline for evaluating the impact of universal HPV vaccination and updated screening strategies.

## 1. Introduction

Human papillomavirus (HPV) is a non-enveloped, icosahedral virion belonging to the *Papillomaviridae* family; its genetic material consists of a single stretch of double-stranded DNA of approximately 8000 bp. It encodes two sets of proteins: the late proteins (L1 and L2) and the early proteins (E1, E2, E4, E5, E6, E7). The late proteins are responsible for the structure of the capsid. The early proteins, especially E5, E6, and E7, govern the integration of viral DNA into the host cell genome, viral DNA replication, and other changes that may lead to the development of cancer [1,2,3,4].

The virus has a high tropism for the epithelial cells of the mucous membranes of the urogenital tract and the oral cavity, as well as for epidermal cells. While it is most often transmitted through sexual contact, there is also a risk of horizontal infection by contact with secretions and blood from infected people, and vertically from mother to child during childbirth [5,6].

Approximately 400 different HPV genotypes have been described. Genotypes with high oncogenicity (including 16, 18, 26, 31, 33, 34, 35, 39, 45, 51, 52, 53, 56, 58, 59, 66, 68, 70, 73) may represent induction factors for cervical cancer, penile cancer, and oropharyngeal cancer. They are typically classified as high-risk genotypes (HR-HPV). Genotypes 6, 11, 40, 42, 43, 44, 54, 61, 72, and 81 are considered low-risk (LR-HPV) and are responsible for the development of benign lesions called condyloma acuminata [4,7].

It is estimated that in the general female population in European countries, HPV16/18 infection occurs at a rate between 2% and 20% in Sweden and Croatia, respectively (https://hpvcentre.net/datastatistics.php (accessed on 21 January 2026). Available data indicate that the prevalence of HPV genotypes in the studied populations of women attending screening varies significantly depending on the region of Europe. For example, HR-HPV types were detected in 12.2% of Spanish women tested for HPV, representing 84% of HPV-positive samples [8]. In Turkey, the prevalence of HR-HPV genotypes was 36.3% [9], while in Central Italy, it was 22.1% [10]. These results are greatly influenced by the vaccination programs implemented in countries. In Danish women, the prevalence of unvaccinated HR-HPV types was 32%, while for vaccinated HPV genotypes, it was reduced to less than 1% [11]. Importantly, across all these populations, the most frequently reported HR-HPV types are 16, 31, 39, 51, 52, and 66 [8,9,10,11]. In Poland, slightly more than half of the women tested for the condition are infected with HPV. The most frequently identified HR-HPV genotypes in this cohort are HPV-16, followed by HPV-31 and HPV-66, while HPV-53 is the most common LR-HPV genotype [12].

As previously mentioned, an important role in preventing the spread of HPV viruses is played by vaccines. Currently, the following vaccines are available in Europe: bivalent (against genotypes 16 and 18), quadrivalent (against genotypes 6, 11, 16, and 18), and nine-valent (against genotypes 6, 11, 16, 18, 31, 33, 45, 52, and 58) [2]. They are intended for adults and children over the age of nine years. In countries where vaccination coverage approached 90%, which was made possible by the implementation of school vaccination programs for both girls and boys, the number of HPV-related cancers detected decreased [13].

Historically, the first recommendations for HPV vaccination were issued in Poland in 2008. It concerned girls aged 11–12. Further recommendations appeared in 2022 and concerned both girls and boys over the age of 9. Neither the 2008 nor the 2022 recommendations mentioned funding; the vaccinations were paid for and available at primary care facilities. Periodically, some local authorities organized HPV vaccination funding programs, but these were limited in time and regional in nature. In all cases, bivalent, quadrivalent, and nonvalent vaccines were available [14].

In Poland, a universal vaccination program against HPV was initiated in 2023 [15]. Government data from 2020 report the diagnosis of cervical cancer in 3800 patients, of which 99% were HPV-related cancers. In the same year, cervical cancer was implicated in 2137 deaths. This large number is primarily due to diagnosis often occurring at a very advanced stage of the disease [16]. Late diagnosis is often caused by low awareness of prevention: indeed, in one study, 68% of respondents reported having only an intermediate level of knowledge on this subject. Furthermore, 39% indicated reluctance to perform cytological tests due to fear of pain. Lack of participation in non-invasive screening tests, e.g., detection of HPV DNA and gynecological cytology, is believed to be a key reason for the spread of HPV infection in Polish society [7].

The aim of the present epidemiological study is to determine the prevalence of HPV infection in the Polish population before the introduction of a universal HPV vaccination program for boys and girls aged 12–13; this period also predated an update of the recommendations regarding cervical cancer screening among female patients. It also identifies most common HPV genotypes in specific groups with regard to age and sex, and examines the potential for co-infection, i.e., the co-occurrence of different HPV genotypes in a single patient.

## 2. Results

### 2.1. Differences in HPV Genotype Distribution Between Males and Females

**Figure 1:**
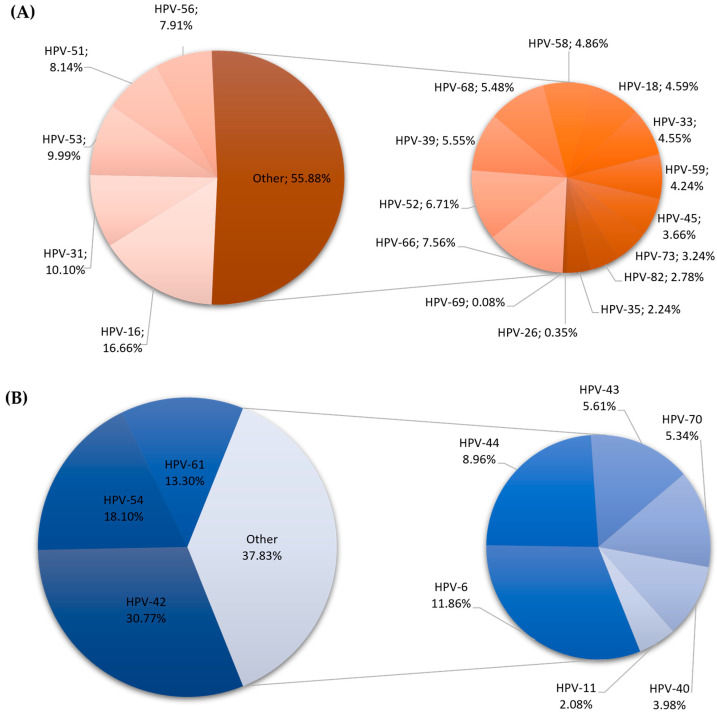
Distribution of high (**A**)- and low (**B**)-risk HPV genotypes in the female population.

**Figure 2:**
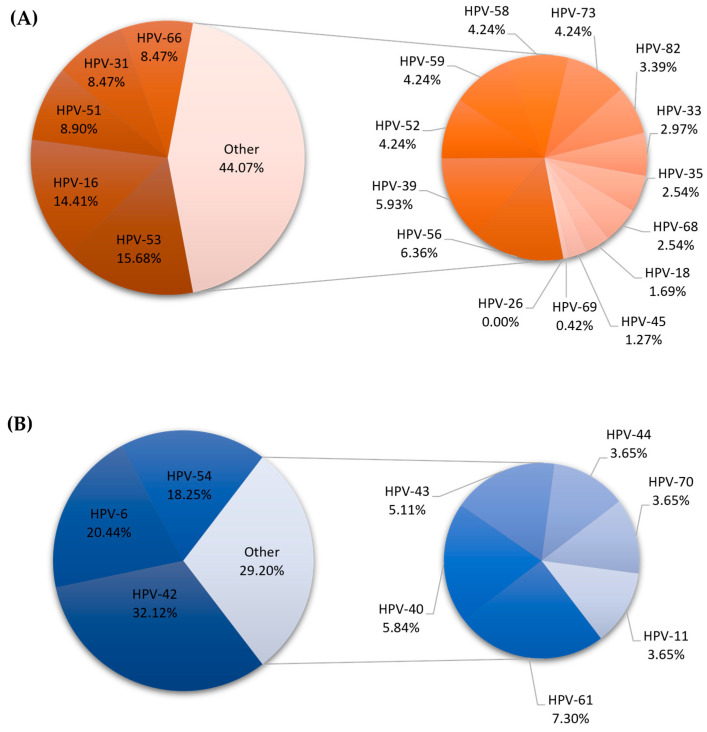
Distribution of high (**A**)- and low (**B**)-risk HPV genotypes in the male population.

Among the 2296 results analyzed, 64.53% were classified as high-risk genotypes and 35.47% as low-risk. The most common high-risk genotypes were HPV-16 (*n* = 466; 15.26%), HPV-53 (*n* = 296; 9.69%), HPV-31 (*n* = 282; 9.23%), and HPV-51 (*n* = 232; 7.60%). The predominant low-risk genotypes were HPV-42 (*n* = 384; 30.92%), HPV-54 (*n* = 225; 18.12%), HPV-6 (*n* = 159; 12.80%), and HPV-61 (*n* = 157; 12.64%). The prevalence of HPV genotypes according to sex is given in Figure 1 (women) and Figure 2 (men).

Regarding the sex distribution of each specific HPV genotype, HPV-18 was detected in 119 cases among 2110 HPV-positive women. This relationship was statistically significant (*p* = 0.0430). Furthermore, infection with HPV-53 (*p* = 0.0030; *n* = 37), HPV-6 (*p* < 0.001; *n* = 28) and HPV-42 (*p* = 0.0080; *n* = 44) was significantly more common in men.

### 2.2. Age-Dependent Patterns of HPV Infection

**Figure 3:**
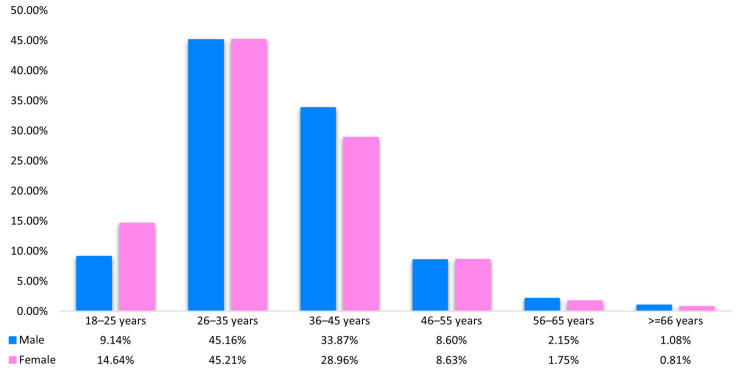
Percentage distribution of HPV-positive results in women and men depending on patient age.

The prevalence of HPV genotypes was also analyzed with regard to patient age. The findings are presented in Figure 3.

Infection was significantly more likely among younger age groups. The highest number of cases was detected in the 26–35 age group, for both men (*n* = 84, 45.16%) and women (*n* = 954, 45.21%). In addition, the prevalence of specific HPV genotypes was compared with patient age (Table 1).

**Figure 4:**
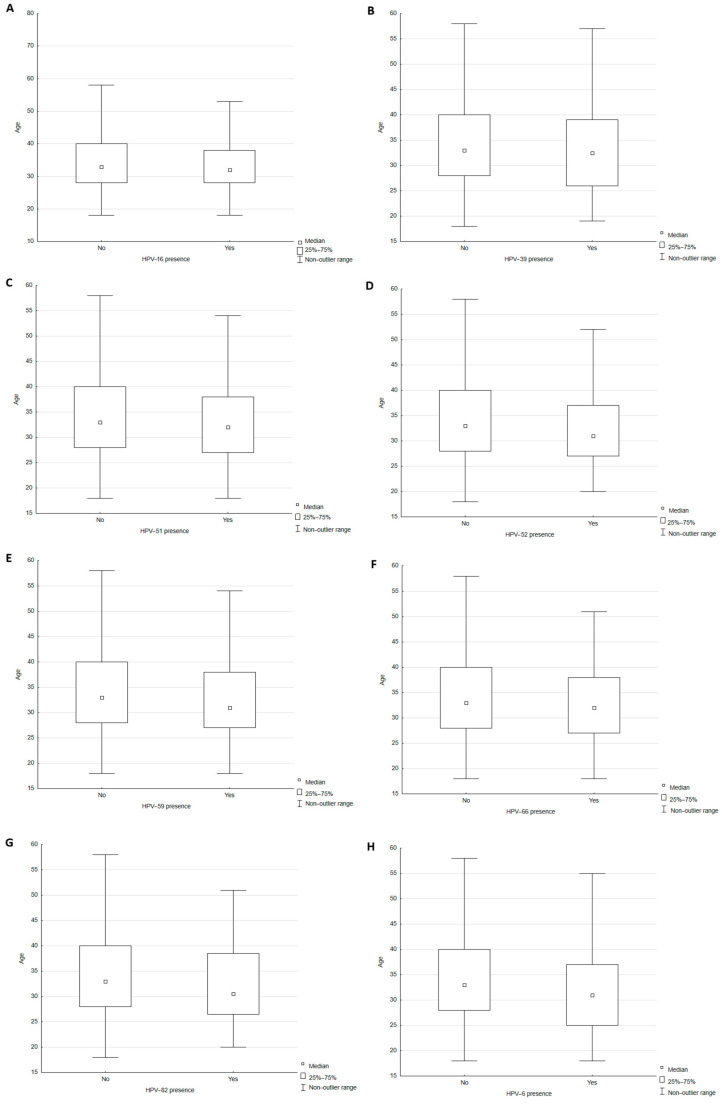
Analysis of the association between patient age and detected HPV-16 ((**A**); Mann–Whitney U *p* = 0.0100), HPV-39 ((**B**); Mann–Whitney U *p* = 0.0190), HPV-51 ((**C**); Mann–Whitney U *p* = 0.0270), HPV-52 ((**D**); Mann–Whitney U *p* < 0.001), HPV-59 ((**E**); Mann–Whitney U *p* = 0.0120), HPV-66 ((**F**); Mann–Whitney U *p* = 0.0150), HPV-82 ((**G**); Mann–Whitney U *p* = 0.0460) and HPV-6 ((**H**); Mann–Whitney U *p* < 0.001).

Some specific virus genotypes were found to be significantly more common in younger patients, based on age at the time of HPV testing (Table 2, Figure 4A–H).

### 2.3. Gender, Age, and Infection Intensity

The number of cases of infection with particular HPV genotypes and their intensity (low, medium, and high) according to sex are summarized in Table 3 (for women) and in Table 4 (for men). A significant association was observed between sex and the intensity of infection with the high-risk genotypes HPV-16, HPV-31, HPV-51, HPV-53, and HPV-56.

Of these, HPV-16 (*p* = 0.0150), HPV-31 (*p* = 0.0360), HPV-51 (*p* < 0.001), and HPV-56 (*p* = 0.0100) were significantly more prevalent at low intensity in men (*p* = 0.0150) than women. In the female population, these genotypes were more frequently at a medium or high intensity. In women, HPV-53 infection was significantly more likely to be high-intensity (*p* = 0.0040) compared to men, who mainly demonstrated low- and medium-intensity infections.

**Figure 5:**
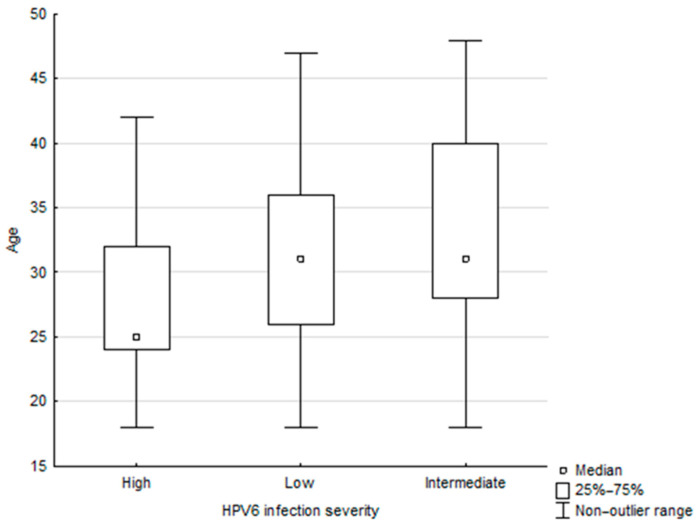
The association between patient age and HPV-6 infection intensity (Kruskal–Wallis *p* = 0.0040).

In addition, the association between patient age and the intensity of infection with particular HPV genotypes was assessed. The only association was demonstrated for HPV-6 (*p* = 0.0040): high-intensity infection was more common in younger patients than medium-intensity infection, while the latter was more common in older patients (Figure 5).

### 2.4. Co-Infection Patterns

**Figure 6:**
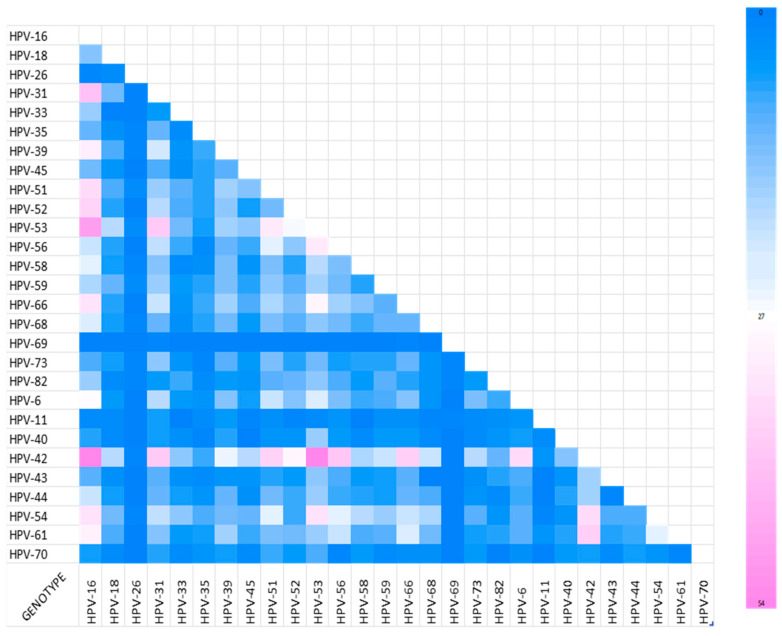
Number of cases of co-infection by pairs of HPV variants.

The frequency of co-infections between the 28 analyzed HPV genotypes is presented in Figure 6.

In the group of 2296 analyzed cases, 1068 individuals were infected with more than one type of HPV variant. The most frequently observed co-infections were HPV-16/42 (*n* = 54, 5.06%), HPV-42/53 (*n* = 54, 5.06%), HPV-16/53 (*n* = 49, 4.59%), and HPV-16/31 (*n* = 41, 3.84%).

### 2.5. Factors Influencing the Number of HPV Genotypes Identified in an Individual Patient

Correlations were observed between the identified HPV genotypes and patient sex, age, and intensity of infection. This data was subjected to multivariate logistic regression analysis (MLRA) to determine whether sex, age, and diagnostic category (NILM, ASCUS, LSIL, HSIL, ASC-H) might prognose the number of HPV genotypes identified in each patient. The results are presented in Table 5.

The results indicate that younger age and cytology result defined as LSIL showed a strong correlation with the presence of higher numbers of HPV genotypes (*p* < 0.001). While male sex also appeared to be associated with a higher number of HPV genotypes (*p* = 0.0183), this significance was lost after post hoc analysis (Bonferroni procedure).

### 2.6. Diagnostic Category of Cytology Result and HPV Type

The cytology result describing the condition of cervical cells was compared with the presence of infection with particular HPV genotypes. Of the 2296 cases collected, data on cytological changes were acquired for 1269. A summary of the changes obtained in liquid-based cytology (LBC) according to the detected HPV genotype is presented in Table 6.

The ASCUS category positively correlates with the presence of high-risk HPV-31 (*p* = 0.0040), HPV-33 (*p* = 0.0080), and HPV-39 (*p* = 0.0390) and low-risk HPV-6 (*p* = 0.0330) and HPV-43 (*p* = 0.0300). The LSIL category was associated with infection of one of two high-risk HPVs, HPV-56 (*p* = 0.0100) and HPV-66 (*p* = 0.0110), or one of two low-risk HPVs, HPV-6 (*p* = 0.0040) and HPV-43 (*p* = 0.0240). The HSIL category was more common during infection with high-risk HPV-16 (*p* < 0.0010) or low-risk HPV-42 (*p* = 0.0470).

## 3. Discussion

The present study provides a comprehensive analysis of the distribution of high-risk (HR) and low-risk (LR) human papillomavirus (HPV) genotypes in a large Polish cohort; it also examines their distribution with regard to patient age, sex, cytological diagnosis, and infection intensity. These findings contribute to the understanding of the epidemiological and biological patterns of HPV infection in Central Europe, with implications for preventive strategies and individualized patient monitoring.

### 3.1. HPV Genotype Distribution and Gender Differences

Among the 2296 analyzed cases, HR-HPVs accounted for nearly two-thirds of infections (64.53%), with HPV-16, HPV-53, HPV-31, and HPV-51 being the most prevalent. This distribution aligns with global data confirming HPV-16 as the dominant oncogenic genotype associated with cervical and anogenital cancers [17]. Interestingly, our data revealed a statistically significant association between female gender and HPV-18 infection, whereas in men, HPV-53, HPV-6, and HPV-42 were more prevalent. These findings suggest potential sex-specific tropisms or behavioral transmission differences. Similar gender-related HPV patterns were observed in a recent Lancet Global Health meta-analysis, which reported that nearly one in three men worldwide is infected with at least one HPV type and one in five with a high-risk genotype [18]. These differences may be related to the anatomical sites of infection, hormonal influences, and variations in immune clearance efficiency [19,20].

The population-level distribution of HPV genotypes observed in our cohort is directly relevant when interpreted against the antigenic composition of currently implemented vaccines. Poland’s universal program offers a choice of the bivalent vaccine (HPV16/18) and the nonvalent vaccine (HPV6/11/16/18/31/33/45/52/58), whereas the earlier quadrivalent formulation (HPV6/11/16/18) is also well characterized in the literature. In our dataset, HPV-16 was the most prevalent HR-HPV genotype (15.26%), and HPV-31—also included in the nonvalent vaccine—ranked among the most frequent types (9.23%), while HPV-18 was detected more often in women. The presence of HPV-6 among the dominant LR-HPV types further supports the expectation that vaccination may reduce not only oncogenic HPV burden but also HPV-associated benign disease. At the same time, several of the most frequent genotypes in this Polish cohort are not vaccine types, including HPV-53 and HPV-51 among high-risk infections and HPV-42 and HPV-54 among low-risk infections. This finding underscores the diagnostic and epidemiological value of broad genotyping assays in the post-vaccination era: continuous surveillance of type-specific prevalence is needed to quantify real-world vaccine impact, to detect potential shifts in genotype distribution, and to guide future refinements of screening and triage algorithms. Given the high proportion of multitype infections in our material, monitoring the dynamics of vaccine versus non-vaccine types may be essential for accurate risk stratification based on genotype combinations and cytological categories.

### 3.2. Age-Dependent Patterns of HPV Infection

Our analysis confirmed that younger patients exhibited a significantly greater prevalence of multiple HPV genotypes, including high-risk types such as HPV-16, HPV-39, HPV-51, HPV-52, HPV-59, HPV-66, and HPV-82, as well as LR-HPV-6. This trend reflects the higher HPV prevalence observed in younger, sexually active populations worldwide [8,21]; the decreasing infection rate observed with age likely results from immune-mediated viral clearance or reduced exposure [22,23,24]. Furthermore, our multivariate logistic regression model demonstrated that age and LSIL cytology category were most strongly associated with a higher number of identified HPV genotypes; these findings confirm previous research emphasizing the role of co-infection in young children in lesion development [25].

### 3.3. Sex and Infection Intensity

A significant association between patient sex and infection intensity was observed for HR genotypes HPV-16, HPV-31, HPV-51, HPV-53, and HPV-56. Men exhibited predominantly low-intensity infections for these variants, whereas women tended to demonstrate higher viral loads. These differences may reflect the influence of hormones on viral replication, or sampling biases related to anatomical sites [19,20]. HPV-53 infections in women tended to be characterized by higher intensity. This may indicate biological persistence rather than higher oncogenic potential: indeed, HPV-53 has previously been found to have a low or uncertain oncogenic risk [26,27].

### 3.4. Co-Infection Patterns and Potential Viral Interactions

Nearly half of the positive samples (46.5%) demonstrated co-infection with multiple HPV types. This finding is consistent with global data indicating a 30–50% prevalence of co-infection [28,29]. The most frequent co-existing pairs in the present study were HPV-16/42, HPV-42/53, HPV-16/53, and HPV-16/31. In contrast, Del Prete et al. report the most frequently occurring pairs to be HPV-42/31, HPV-42/53, HPV-42/6, HPV-53/31, and HPV-54/53 [6]. However, current evidence remains inconsistent: while some studies link multiple HPV infections to increased lesion severity [28,29], others report no clear additive risk for CIN3+ development [30]; this is in line with our present observations that co-infections may not always translate into a greater oncogenic burden.

### 3.5. Cytological Findings and Genotype Correlations

Distinct correlations were identified between cytological diagnosis and specific HPV genotypes. The LSIL category was predominantly linked to HPV-56 and HPV-66, while HSIL correlated with HPV-16 and, unexpectedly, LR-HPV-42. This last observation could reflect co-infection dynamics or misclassification due to viral load differences. Similar findings were reported by Kjær et al. (2014), who observed that certain LR types may persist in lesions alongside HR-HPVs, which may influence cytological outcomes [31]. Moreover, the ASCUS cases in the present study demonstrated a higher prevalence of HPV-31, HPV-33, and HPV-39: HR genotypes are strongly associated with early intraepithelial alterations [31,32].

### 3.6. Clinical and Epidemiological Implications

The observed age- and sex-dependent patterns of HPV genotype prevalence underscore the importance of tailored prevention and screening strategies. The high prevalence of HR-HPVs, especially among younger women with LSIL cytology, supports early vaccination and HPV-based screening, which is consistent with current WHO recommendations [33]. Furthermore, by developing a greater understanding of the intensity of genotype-specific infection and co-infection profiles, it would be possible to refine molecular triage algorithms for cervical cytology, thus improving diagnostic specificity and personalized follow-up.

### 3.7. Limitations and Future Directions

A limitation of the study is its cross-sectional design, with no longitudinal follow-up performed to assess viral persistence and lesion progression. Future research should aim to integrate viral load quantification, host genetic polymorphisms, and immune status to provide a deeper insight into genotype-specific pathogenicity. Additionally, extending molecular analyses to male anogenital and oropharyngeal samples would enhance understanding of HPV epidemiology across both sexes.

## 4. Materials and Methods

### 4.1. Study Population

Anonymized biomedical datasets were acquired retrospectively for analysis. The study group comprised 2296 adult patients (2110 women and 186 men) who had received a positive HPV test result following routine cervical cancer screening or during examination of genital or oral lesions. Among the women (mean age 33 years; range 18–77 years), the study material comprised swabs from the cervical canal and ectocervix/vaginal portion of the cervix, urethral swabs, and oral swabs. For the men (mean age 34 years; range 18–66 years), the material included swabs from the sulcus retroglandularis, oral cavity, and urethra.

Specimen collection was performed at collaborating healthcare facilities providing diagnostic services within Synevo Polska. These facilities included hospitals, outpatient specialist clinics, and individual medical practices, ensuring broad clinical representation of the study population. All specimens were collected using standardized swab-based procedures in accordance with laboratory-approved instructions provided by Synevo Polska, which are uniformly implemented across all cooperating sites. Sample collection was performed exclusively by authorized medical personnel, namely licensed physicians or certified midwives. Self-sampling was not permitted or used in this study. Swabs were obtained from predefined, clinically indicated anatomical sites, including the cervical canal and ectocervix/vaginal portion of the cervix, urethra, and oral cavity, using validated collection devices and transport media. To avoid specimen duplication, each sample was assigned a unique laboratory identifier at the time of collection and registered in the laboratory information system. Duplicate specimens from the same patient and anatomical site were excluded from analysis in accordance with standard laboratory quality assurance procedures. All collection and handling procedures were conducted in compliance with applicable clinical and laboratory standards, ensuring methodological consistency, sample integrity, and reproducibility of results across all participating centers.

The following data were extracted: age, sex, detected HPV genotype, and identified category of cervical epithelial cell abnormalities according to the current classification in gynecological smears. All datasets included in this study were collected in the Laboratory Information System (Marcel S.A., Zielonka, Poland) between August 2022 and March 2023.

The HPV genotyping included in the datasets was performed in the Molecular Biology Laboratory of Synevo Medical Laboratory, Lodz, Poland. The genetic material was isolated using a Hamilton Microlab NIMBUS v1.13 (Seoul, Republic of Korea) automated pipetting station and STARMag 96 × 4 Universal Cartridge Kit (Seoul, Republic of Korea ) extraction reagents. The tests were carried out using Anyplex™II HPV 28 kits (Seoul, Republic of Korea), which detect the presence of 28 HPV genotypes, including 19 high-risk HPVs (16, 18, 26, 31, 33, 35, 39, 45, 51, 52, 53, 56, 58, 59, 66, 68, 69, 73, 82) and nine low-risk HPVs (6,11, 40, 42, 43, 44, 54, 61, 70). The results were read based on the melting curves of real-time PCR products after 30, 40, and 50 reaction cycles. The curves were interpreted automatically in the Seegene Viewer program v3.28.000 (Seoul, Republic of Korea), and the results of the detection of a particular genotype are given in the form of ‘+’ signs, the number of which (from one to three) depends on whether the signal was read in each melting curve run.

The retrospective analysis was approved by the local Ethics Committee (Bioethics Committee of the Medical University of Lodz; decision no. RNN/96/25/KE). Patient consent was waived due to the nature of the study (retrospective cohort study). All identifying information has been removed from the data, and researchers cannot link participants to the data. The Bioethics Committee of the Medical University of Lodz approved the research using existing data from laboratory analyses, in accordance with the study protocol.

### 4.2. Statistical Analysis

The statistical analysis was performed using Statistica 13.3 software (TIBCO, Palo Alto, CA, USA). The Shapiro–Wilk test was used to confirm the normality of continuous distributions. The Mann–Whitney U-test was used to compare pairs of groups, and the Kruskal–Wallis one-way analysis of variance by ranks to compare more than two groups.

The frequency of a particular HPV genotype was compared with other categorical parameters by constructing a two-way contingency table to display the relationship between two categorical variables. Pearson’s chi-squared test was used to establish statistical significance.

To analyze the relationship between a single dependent variable and one or more independent variables, regression models were built using categorical (factors) and/or continuous (covariates) predictors. The post hoc analysis was performed using the Bonferroni correction where applicable (FDR 1%).

In all subsequent calculations, the significance was set at *p* < 0.05.

## 5. Conclusions

Our data confirm a high prevalence of HPV in the female and male populations in Poland. This prevalence is particularly high among patients aged 26–35, confirming the validity of lowering the testing age to 25. Nevertheless, the recently introduced universal vaccination program for children aged 12–13 and changes to the diagnostic scheme for cervical cancer screening appear to be achieving positive effects.

Most importantly, our findings emphasize that LR-HPV genotyping should be included in screening programs. Currently, full HPV genotype panels are not mandatory; however, their use should be expanded to identify variants with carcinogenic potential, such as HPV-42, to allow for earlier detection of cervical cancer. Despite its current status as a low-risk genotype, it may possess potential carcinogenicity and should be closely monitored.

We hope that the nationwide vaccination program will reduce the observed frequency of HPV infections in the following years. Nonetheless, both the program and its findings should be mandatorily monitored to cease the spread of HPV infections among people from Poland and, through this, reduce HPV-related cancers. Subsequent data collection should also aim to answer the question of whether there is also a need for broader genotyping as well as a new, broader vaccination program.

## Figures and Tables

**Table 1:** Distribution of HPV genotypes in particular age groups of the studied population.

HPV Genotype		Age Group					
		18–25 Years Number of Cases (%)	26–35 Years Number of Cases (%)	36–45 Years Number of Cases (%)	46–55 Years Number of Cases (%)	56–65 Years Number of Cases (%)	>66 Years Number of Cases (%)
High Risk Genotypes	HPV-16	80 (11.10%)	220 (11.54%)	121 (9.96%)	35 (10.17%)	8 (12.12%)	2 (5.56%)
	HPV-18	22 (3.05%)	57 (2.99%)	30 (2.47%)	9 (2.62%)	3 (4.55%)	2 (5.56%)
	HPV-26	0	6 (0.31%)	3 (0.25%)	0	0	0
	HPV-31	30 (4.16%)	135 (7.08%)	78 (6.42%)	25 (7.27%)	3 (4.55%)	3 (8.33%)
	HPV-33	19 (2.64%)	61 (3.20%)	36 (2.96%)	3 (0.87%)	4 (6.06%)	2 (5.56%)
	HPV-35	11 (1.53%)	26 (1.36%)	19 (1.56%)	3 (0.87%)	3 (4.55%)	2 (5.56%)
	HPV-39	38 (5.27%)	63 (3.31%)	45 (3.70%)	9 (2.62%)	3 (4.55%)	0
	HPV-45	10 (1.39%)	43 (2.26%)	35 (2.88%)	8 (2.33%)	2 (3.03%)	0
	HPV-51	44 (6.10%)	101 (5.30%)	64 (5.27%)	19 (5.52%)	1 (1.52%)	3 (8.33%)
	HPV-52	36 (4.99%)	92 (4.83%)	41 (3.37%)	14 (4.07%)	1 (1.52%)	0
	HPV-53	58 (8.04%)	117 (6.14%)	86 (7.08%)	30 (8.72%)	2 (3.03%)	3 (8.33%)
	HPV-56	39 (5.41%)	97 (5.09%)	58 (4.77%)	17 (4.94%)	4 (6.06%)	5 (13.89%)
	HPV-58	24 (3.33%)	58 (3.04%)	38 (3.13%)	12 (3.49%)	4 (6.06%)	0
	HPV-59	22 (3.05%)	62 (3.25%)	25 (2.06%)	7 (2.08%)	4 (6.06%)	0
	HPV-66	39 (5.41%)	101 (5.30%)	57 (4.69%)	14 (4.07%)	4 (6.06%)	1 (2.78%)
	HPV-68	16 (2.22%)	70 (3.67%)	45 (3.70%)	15 (4.36%)	2 (3.03%)	0
	HPV-69	1 (0.14%)	1 (0.05%)	0	1 (0.29%)	0	0
	HPV-73	14 (1.94%)	38 (1.99%)	34 (2.80%)	7 (2.03%)	1 (1.52%)	0
	HPV-82	14 (1.94%)	41 (2.15%)	17 (1.40%)	7 (2.03%)	1 (1.52%)	0
Low Risk Genotype	HPV-6	41 (5.69%)	69 (3.62%)	35 (2.88%)	12 (3.49%)	1 (1.52%)	1 (2.78%)
	HPV-11	7 (0.97%)	8 (0.42%)	10 (0.82%)	1 (0.29%)	2 (3.03%)	0
	HPV-40	10 (1.39%)	20 (1.05%)	16 (1.32%)	5 (1.45%)	1 (1.52%)	0
	HPV-42	73 (10.12%)	151 (7.92%)	117 (9.63%)	35 (10.17%)	2 (3.03%)	6 (16.67%)
	HPV-43	10 (1.39%)	36 (1.89%)	16 (1.32%)	6 (1.74%)	1 (1.52%)	0
	HPV-44	12 (1.66%)	42 (2.20%)	36 (2.96%)	12 (3.49%)	0	2 (5.56%)
	HPV-54	25 (3.47%)	109 (5.72%)	65 (5.35%)	20 (5.81%)	5 (7.58%)	1 (2.78%)
	HPV-61	21 (2.91%)	59 (3.10%)	59 (4.86%)	12 (3.49%)	3 (4.55%)	3 (8.33%)
	HPV-70	5 (0.69%)	23 (1.21%)	29 (2.39%)	6 (1.74%)	1 (1.52%)	0

**Table 3:** Prevalence of individual HPV genotypes and intensity of infection in women.

Genotype	Number of Cases for a Given Intensity of Infection						Number of Cases in Women
	Low Intensity		Medium Intensity		High Intensity		
HPV-16	97	22.45%	223	51.62%	112	25.93%	432
HPV-18	19	15.97%	64	53.78%	36	30.25%	119
HPV-26	4	44.44%	4	44.44%	1	11.11%	9
HPV-31	73	27.86%	135	51.53%	54	20.61%	262
HPV-33	22	18.64%	65	55.08%	31	26.27%	118
HPV-35	8	13.79%	36	62.07%	14	24.14%	58
HPV-39	39	27.08%	71	49.31%	34	23.61%	144
HPV-45	33	34.74%	55	57.89%	7	7.37%	95
HPV-51	46	21.80%	109	51.66%	56	26.54%	211
HPV-52	52	29.89%	97	55.75%	25	14.37%	174
HPV-53	45	17.37%	125	48.26%	89	34.36%	259
HPV-56	60	29.27%	93	45.37%	52	25.37%	205
HPV-58	24	19.05%	63	50.00%	39	30.95%	126
HPV-59	46	41.82%	44	40.00%	20	18.18%	110
HPV-66	62	31.63%	81	41.33%	53	27.04%	196
HPV-68	61	42.96%	60	42.25%	21	14.79%	142
HPV-69	1	50.00%	1	50.00%	0	0.00%	2
HPV-73	41	48.81%	28	33.33%	15	17.86%	84
HPV-82	23	31.94%	35	48.61%	14	19.44%	72
HPV-6	41	31.30%	55	41.98%	35	26.72%	131
HPV-11	5	21.74%	7	30.43%	11	47.83%	23
HPV-40	15	34.09%	20	45.45%	9	20.45%	44
HPV-42	79	23.24%	179	52.65%	82	24.12%	340
HPV-43	25	40.32%	28	45.16%	9	14.52%	62
HPV-44	33	33.33%	49	49.49%	17	17.17%	99
HPV-54	97	48.50%	93	46.50%	10	5.00%	200
HPV-61	45	30.61%	76	51.70%	26	17.69%	147
HPV-70	15	25.42%	31	52.54%	13	22.03%	59

**Table 4:** Prevalence of individual HPV genotypes and intensity of infection in men.

Genotype	Number of Cases for a Given Intensity of Infection						Number of Cases in Men
	Low Intensity		Medium Intensity		High Intensity		
HPV-16	15	44%	14	41%	5	15%	34
HPV-18	0	0%	3	75%	1	25%	4
HPV-26	0	-	0	-	0	-	0
HPV-31	11	55%	7	35%	2	10%	20
HPV-33	2	29%	3	43%	2	29%	7
HPV-35	0	0%	6	100%	0	0%	6
HPV-39	4	29%	9	64%	1	7%	14
HPV-45	1	33%	2	67%	0	0%	3
HPV-51	13	62%	6	29%	2	10%	21
HPV-52	4	40%	5	50%	1	10%	10
HPV-53	13	35%	20	54%	4	11%	37
HPV-56	10	67%	4	27%	1	7%	15
HPV-58	4	40%	6	60%	0	0%	10
HPV-59	7	70%	3	30%	0	0%	10
HPV-66	8	40%	10	50%	2	10%	20
HPV-68	2	33%	4	67%	0	0%	6
HPV-69	0	0%	0	0%	1	100%	1
HPV-73	4	40%	5	50%	1	10%	10
HPV-82	5	63%	3	38%	0	0%	8
HPV-6	8	29%	14	50%	6	21%	28
HPV-11	2	40%	2	40%	1	20%	5
HPV-40	1	13%	5	63%	2	25%	8
HPV-42	9	20%	26	59%	9	20%	44
HPV-43	2	29%	3	43%	2	29%	7
HPV-44	0	0%	4	80%	1	20%	5
HPV-54	11	44%	13	52%	1	4%	25
HPV-61	5	50%	3	30%	2	20%	10
HPV-70	1	20%	4	80%	0	0%	5

**Table 5:** Multivariate logistic regression model analyzing the effect of age at the time of sample collection, cytology result, and sex on the number of identified HPV genotypes. NILM—negative for intraepithelial lesions or malignancy; ASCUS—atypical squamous cells of undetermined significance; LSIL—low-grade squamous intraepithelial neoplasia; HSIL—high-grade squamous intraepithelial neoplasia; ASC-H—atypical squamous cell cannot exclude HSIL.

	Factors	*p*-Value
Number of identified HPV genotypes	Age	<0.001
	
	Diagnostic category	
	NILM	0.7506
	ASCUS	0.1968
	LSIL	<0.001
	HSIL	0.2779
	ASC-H	0.0839
	
	Sex	0.0183

**Table 6:** Number of cases of changes in liquid-based cytology depending on the detected HPV genotype.

Genotypes		LIQUID-BASED CYTOLOGY CHANGES				
		NILM	ASCUS	LSIL	HSIL	ASCH
**High-Risk Genotypes**	HPV-16	2	51	97	37	10
	HPV-18	0	18	29	6	2
	HPV-26	0	0	1	0	0
	HPV-31	1	52	56	10	4
	HPV-33	0	26	26	6	1
	HPV-35	0	4	15	0	2
	HPV-39	1	29	36	4	2
	HPV-45	0	16	19	3	0
	HPV-51	0	22	51	9	2
	HPV-52	0	31	35	7	2
	HPV-53	0	29	75	5	6
	HPV-56	0	24	55	4	3
	HPV-58	0	19	26	5	2
	HPV-59	0	15	24	2	4
	HPV-66	1	32	54	2	3
	HPV-68	0	17	31	4	3
	HPV-69	0	0	0	0	0
	HPV-73	0	17	17	1	4
	HPV-82	0	7	17	0	1
**Low-Risk Genotypes**	HPV-6	0	12	16	4	1
	HPV-11	0	2	3	0	0
	HPV-40	0	7	13	0	1
	HPV-42	0	56	69	7	2
	HPV-43	0	3	20	3	1
	HPV-44	0	17	19	2	1
	HPV-54	0	33	40	6	1
	HPV-61	0	22	32	4	0
	HPV-70	0	8	10	2	2

**Table 2:** Individual HPV genotypes that were found to be significantly more prevalent in younger age groups.

Genotype		*p*-Value
High-Risk Genotypes	HPV-16	0.0100
	HPV-39	0.0190
	HPV-51	0.0270
	HPV-52	<0.001
	HPV-59	0.0120
	HPV-66	0.0150
	HPV-82	0.0460
Low-Risk Genotypes	HPV-6	<0.001

## Data Availability

Dataset available on request from the authors.

## Abbreviations

The following abbreviations are used in this manuscript:

HPV	Human Papillomavirus
HR-HPV	High-Risk Human Papillomavirus
LR-HPV	Low-Risk Human Papillomavirus
NILM	Negative for Intraepithelial Lesion or Malignancy
ASCUS	Atypical Squamous Cells of Undetermined Significance
LSIL	Low-Grade Squamous Intraepithelial Lesion
HSIL	High-Grade Squamous Intraepithelial Lesion
ASC-H	Atypical Squamous Cells—Cannot Exclude High-Grade SIL
CIN	Cervical Intraepithelial Neoplasia
WHO	World Health Organization
